# Book Review: *Teucrium Species: Biology and Applications*; Stanković, M., Ed.; Springer Nature: Cham, Switzerland, 2020; ISBN: 978-3-030-52158-5

**DOI:** 10.3390/plants11010106

**Published:** 2021-12-30

**Authors:** Pierre Meerts, Christophe Hano

**Affiliations:** 1 Département de Biologie des Organismes, Faculté des Sciences, Université Libre de Bruxelles, Av. F.D. Roosevelt 50 CP 244, 1050 Bruxelles, Belgium; 2 Laboratoire de Biologie des Ligneux et des Grandes Cultures, INRA USC1328, Eure & Loir Campus, Orleans University, 28000 Chartres, France

The genus *Teucrium* is one of the most species-rich and widespread genera of the Lamiaceae family. Species of this genus are mostly perennial, rarely annual, shrubby, sub-shrubby, or herbaceous plants distributed in various habitats, mostly thermophilic or temperate, in the Mediterranean and surrounding areas. *Teucrium* species are an interesting object of research both in basic science, including systematics, taxonomy, ecology, and in applied science, including pharmacy, food and beverage industry. Moreover, medicinal species of this genus known as germanders have a long tradition of use. *Teucrium* species are rich in secondary metabolites with different therapeutic and biological effects. For these reasons, the book “*Teucrium* species: Biology and applications” (ISBN 978-3-030-52158-5), as a comprehensive review, covers and discusses all of the above aspects. The book contains 15 chapters that encompass recent advances in exploring the unique features of *Teucrium* species, including morphology, systematics, biogeography, ecology, ethnobotany, phytochemistry, and biological activity of secondary metabolites, as well as current challenges and further perspectives in the application.

Teresa Navarro [1] proposes a very synthetic review of the systematics and biogeography of *Teucrium* [1]. The chapter includes an interesting historical perspective of the taxonomic concepts of the genus (434 taxa). The chapter is based on original data from 341 herbarium specimens of 97 taxa from throughout the world. Relationships with closely related genera (*Spartothamnella*, *Oncinocalyx*, *Teucridium*, *Rubiteucris,* and *Leucosceptrum*) receive much attention. The chapter comprises a checklist of all accepted *Teucrium* species and intraspecific taxa. The chapter is nicely illustrated by 25 color photographs of selected *Teucrium* species.

The morphological characteristics of *Teucrium* species are presented in two separate chapters. A chapter devoted to the morphology of vegetative organs addresses variation in vegetative traits, including life forms [2]. Particular attention is paid to variation in leaf shape. Micromorphological characters are also examined, including trichome micromorphology. Here, SEM (scanning electron microscope) pictures should have been included to illustrate the different types of trichomes. Functional significance of trichome variation could have been more extensively discussed in relation to habitat type. A chapter dealing with reproductive morphology explores variation patterns in reproductive traits both in a systematic and biological perspective [3]. Much emphasis is laid on the comparison with closely related genera. The adaptive significance of floral trait variation and correlations between floral traits and pollination systems are also discussed. The chapter comprises much information on floral trait variation across infrageneric taxa, presented in the form of very synthetic tables. Trait mapping on a recently published phylogeny would have given more insight into the evolutionary significance of floral trait variation. Finally, fruit traits are characterized, and their significance to the dispersal system is discussed.

Chapter 4 reviews the ecophysiology of two selected *Teucrium* species, i.e., *T. chamaedrys* and *T. montanum* [4]. Both species have a very broad niche. The chapter is focused on intraspecific variation in relation to soil chemical properties. We found the comparison of populations on and off serpentine soil particularly interesting. The genetic and environmental factors that determine the variation in element concentrations in the plants are examined. Some attention is also paid to the secondary metabolites and morphological traits as influenced by the serpentine environment. A table summarizing the variation range of elemental concentrations in populations of both species from contrasting soil types would have been useful.

*Teucrium* species are rich in a range of secondary metabolites with important biological functions. Ethnobotany is the main source for many studies on the biological activity and phytochemical composition of plant extracts and essential oils, as well as biotechnological applications of *Teucrium* species. The medicinal properties of *Teucrium* species can be attributable to their chemical components, notably essential oils, phenolic acids, flavonoids, and other secondary metabolites, according to the rich ethnobotanical literature on *Teucrium* species, which is described in a comprehensive chapter [5]. Two chapters describe the phytochemistry and secondary metabolites diversity of the genus *Teucrium* [6,7]. *Teucrium* species have been shown as a rich source of phenolic acids, phenylethanoids, and flavonoids, but flavonoids are by far the most chemotaxonomically significant for this genus. To expand knowledge and give sufficient data for future research on phenolic compounds of this genus, biosynthetic pathways, as well as extraction methods, are also discussed [6]. *Teucrium* species are rich in essential oils and numerous physiologically active monoterpenoid and sesquiterpenoid compounds, summarized to provide an overview of their compositions, with special attention to the more abundant components and known biological activities, which often confirm the traditional and folk uses of this genus. However, the need for further research into the factors that drive chemical polymorphism in essential oils and, as a result, their bioactivity is pointed [7]. 

However, the authors of this chapter point out that long-term use of some of the preparations can result in undesirable side effects, such as liver inflammation, hepatotoxicity, or a gradual loss of neuromuscular coordination, induced in particular by certain types of diterpenes. To promote the safe use of some species, these side effects and precautions are fully detailed [8]. Antimutagenic properties possibly related to their high concentration of polyphenolic compounds are also fully described [9]. Interestingly, some investigated *Teucrium* species may have a genotoxic effect at high concentrations but showed a protective effect when combined with known mutagenics at the same concentrations. As a result, these extracts could be a potential source of new pharmaceutics that will have a significant protective effect against chemotherapeutic-induced genetic damages.

These species are widely used to treat digestive and respiratory problems, abscesses, gout, and conjunctivitis, as well as fat and cellulite breakdown. They also have anti-inflammatory, antioxidant, anticancer, antibacterial, antidiabetic, and anthelminthic qualities. A comprehensive review of their antioxidant activity in relation to their secondary metabolites content and diversity is proposed [10]. The promising antiviral activity of *Teucrium* species is presented, which may open up a new field of research to aid in the control of plant virus infections [11]. Although a large database of antibacterial characteristics of *Teucrium* species has been compiled over the last 20 years, the mechanisms of activity are still unknown. As pointed by the authors, only a thorough understanding of their bioavailability, pharmacodynamics, and modes of action, according to the author, would allow for the development of a new generation of standardized, successful biopreparations [12]. An overview of existing in vitro and in vivo evidence supporting the anticancer potential is also presented, and the comparative analysis of results may contribute to a better understanding of probable mechanisms of action and encourage future studies into the *Teucrium* species potential as adjuvant chemotherapeutics [13]. A comprehensive examination of how *Teucrium* species high amounts of flavonoids, phenolic acids, and terpenoids directly contribute to anticholinesterase, anti-diabetic, and anti-inflammatory activity, making them attractive candidates for possible future applications, is also presented [14]. All of these biological activities make *Teucrium* species attractive candidates for a range of biotechnological approaches to improve their exploitation while also guaranteeing long-term conservation and wise use of biodiversity, which are also thoroughly discussed in the last chapter [15].

To summarize, the editor has clearly done a great job of bringing together experts from various disciplines to provide the most comprehensive view of current research results and their application on *Teucrium* species. This book is an indispensable reference as it covers all aspects of the plant genus *Teucrium*. It includes a complete bibliography that brings together the most up-to-date knowledge in all disciplines of biology, biotechnology, and pharmacy, as well as more practical information (such as an up-to-date list of species, compounds, and other information) on the *Teucrium* genus. This book is an essential review of the literature for beginners, as well as experts, such as practitioners involved in medicinal plant applications. It can also be used as a university-level literature course for students or PhD students interested in *Teucrium* species, phytochemistry, or other biological activities and uses of plants. To be recommended without any reservations.
